# Characterization of Monoclonal Antibodies against σA Protein and Cross-Reactive Epitope Identification and Application for Detection of Duck and Chicken Reovirus Infections

**DOI:** 10.3390/pathogens8030140

**Published:** 2019-09-07

**Authors:** Xueming Chen, Tongtong Li, Xiaodan Chen, Chenxi Li, Weiwei Lin, Hongyu Liu, Shuping Song, Xiaofei Bai, Yun Zhang

**Affiliations:** State Key Laboratory of Veterinary Biotechnology, Harbin Veterinary Research Institute of Chinese Academy of Agricultural Sciences, Harbin 150069, China (X.C.) (T.L.) (X.C.) (C.L.) (W.L.) (H.L.) (S.S.) (X.B.)

**Keywords:** duck reovirus, avian reovirus, turkey reovirus, monoclonal antibody, σA protein epitopes, cross-reactive epitopes, diagnosis

## Abstract

Although σA is an important major core protein of duck reovirus (DRV), the B-cell epitopes of this protein remain unknown to reseacrhers. Six monoclonal antibodies (MAbs) (1A7, 3F4, 5D2, 4E2, 3C7, and 2B7) were developed by using prokaryotic-expressed recombinant His-σA protein. Five of six MAbs (1A7, 3F4, 4E2, 3C7, and 2B7) reacted with His-σA protein in a conformation-independent manner, while 5D2 reacted with σA in a conformation-dependent manner. Immunofluorescence assays showed that the MAbs could specifically bind to DRV infected BHK-21 cells. The MAbs were delineated as three groups by a competitive binding assay. By using 12-mer peptide phage display and mutagenesis, MAb 4E2 was identified to recognize minimal epitope ^56^EAPYPG^61^ and MAb 1A7 recognize ^341^WVV/MAGLI/V^347^, residues ^341^V/M and ^347^I/V are replaceable. Dot blotting and sequence analysis confirmed that EAPYPG and WVV/MAGLI/V are cross-reactive epitopes in both DRV and avian reovirus (ARV). An enzyme-linked immunosorbent assay (ELISA) based on two expressed EAPYPG and WVVAGLI as antigen demonstrated its diagnostic potential by specific reacting with serum samples from DRV- or ARV-infected birds. Based on these observations, an epitope-based ELISA could be potentially used for DRV or ARV surveillance. These findings provide insights into the organization of epitopes on σA protein that might be valuable for the development of epitope-based serological diagnostic tests for DRV and ARV infection.

## 1. Introduction 

Duck reovirus (DRV) is a member of genus Orthoreovirus in the family of Reoviridae. The viral genome contains ten double-stranded RNA segments, including three size classes: large (L1–L3), medium (M1–M3) and small (S1–S4), which encode eight structural proteins (λA, λB, λC, μA, μB, σA, σB, and σC) and several nonstructural proteins (μNS, σNS, p10.8, etc.) [1,2,3,4,5,6,7,8]. Two types of duck reovirus have been identified: classical DRV (C-DRV) and new DRV (N-DRV). The C-DRV and N-DRV isolates share identical properties in growth properties and genome organization [2,3,4,7,9,10,11], but are different in pathology. The C-DRV is an important Muscovy duckling pathogen involved in viral arthritis/tenosynovitis, growth retardation, myocarditis, enteritis, hepatitis, bursal and thymic atrophy, osteoporosis, respiratory syndromes, and sudden death [12,13,14]. The C-DRV could cause high morbidity and up to 50% mortality in Muscovy ducklings, and recovered birds are often stunted in growth [5]. The N-DRV caused the disease characterized mainly by hemorrhagic-necrotic lesions in the liver and spleen of ducklings [9,10]. The N-DRV could cause duckling death (as early as 5-days-old) with mortality in the range of 10–15%. Chicken reovirus (named avian reovirus, ARV) has been associated with diseases of viral arthritis and pale bird syndrome in chicken [15,16,17,18]. DRV and ARV are antigenically different [2,5,6,9,15,16,17,18] and their cell-attachment protein σC showed only 21–25% identity at amino acid levels [2,7,16,17,18,19,20]. 

The virion major core proteins σ2 of mammalian reovirus (MRV) or σA of ARV and DRV have limited sequence diversity compared to their other S-class proteins [7,20,21,22]. Although the σA proteins were encoded by S1 gene of DRV and S2 genes of ARV, turkey reovirus (TRV), and goose reovirus (GRV), respectively [7,23,24], the secondary structure of DRV σA was very similar to that of ARV or σ2 of MRVs [7,22,23], indicating that they might have similar function [3,7,24]. The crystal structure of recombinant σA of ARV has been successfully reported [25], which confirmed that dsRNA binded to the inside of self-assembled helix. The minimal 14 to 18 bp of double-stranded RNA was required for σA binding.

Since σA-encoding gene between DRV and ARV showed less divergence (about 76.0–77.1% identity), it was often targeted for molecular diagnostic analysis for bird reoviruses (ARV, DRV, GRV, and TRV) infection [13,26,27,28,29]. Thus, based on the premise of highly homologous common immunogenic regions, we explored to develop cross-reactive σA-specific MAbs against both DRV and ARV.

The identification of epitopes for viral proteins can provide important information for understanding of immunological responses and pathogenesis in virus infection. In the case of DRV, efforts have been made to map the epitopes of σB protein [30,31], but epitopes of σA have rarely been reported and the antigenic information for DRV σA is still not defined.

Because of high sensitivity and easy operation, traditional serological ELISA diagnosis has been the principle consideration in laboratory. But the disadvantages of using homologous antigen for accurate detection of antibody and presence of non-specific antigen causing false-negative results, it need to standardize the antigen each time. Therefore, alternative serological tests would be necessary to target virus specific antigenic epitopes to avoid the disadvantages. Many epitope-based enzyme immunoassays have been successfully developed for the detection of virus-specific antibodies in serum samples [32,33,34,35]. Therefore, for increasing the specificity of the diagnosis method, screening of single molecules with potential as diagnosis antigen should be explored.

In this study, six MAbs against the σA protein of DRV were produced and characterized. We identified two minimal σA protein epitopes and assessed their cross-reactivity to DRV and ARV. An ELISA based on expressed epitopes as antigens was evaluated to detect antibodies in serum samples from DRV- and ARV-infected birds. These findings will extend our understanding to σA protein structure–function relationships and provide insights into the improvement of the birds reovirus serodiagnosis and the understanding of the viral pathogenesis.

## 2. Materials and Methods

All experiments involving animals were approved by the Animal Welfare and Ethical Censor Committee at Harbin Veterinary Research Institute (HVRI). All animal experiments were approved by the Animal Ethics Committee of the HVRI of the Chinese Academy of Agricultural Sciences with license SYXK (Heilongjiang) 2011022.

### 2.1. Virus and Sera

Muscovy duck reovirus S12 was propagated in duck embryo or embryo fibroblasts (DEF) or BHK-21 cells as described previously [5,7,22]. The sera of duck anti-DRV sera, chicken anti-ARV, duck/chicken anti-H5N1 influenza virus, duck/chicken anti-new castle disease virus, duck anti-Tembusu virus, and duck anti-parvovirus were described previously [7,31].

### 2.2. Expression of σ A Protein

Viral RNA was extracted from DRV by using an Easyspin RNA Extraction Kit (Qiagen, Shanghai, China). AMV reverse transcriptase (TaKaRa, Dalian, China) was used to reverse-transcribe the RNAs into cDNAs with a random hexomer oligonucleotide primer as described previously [4]. Primers to σA-encoding gene PAF: 5′-TTTGAATTCATGGCGCGTGCCGTGTACGACTTCTT-3′ (*Eco*RI site is underlined) and PAR: 5′-GTCTCGAGCTAGACGGTAAAAGTGGC-3′ (*Xho*I site is underlined) were designed using Oligo 6.24 (Molecular Biology Insights, Inc. Beijing, China) based on homologous nucleotide sequences from previously reported DRV. σA-encoding gene was amplified from the cDNA by PCR using primers PAF and PAR. The amplified cDNA were separated on a 1% agarose gel and purified with gel extraction kit (Qiagen Inc., Valencia, CA, USA). The purified PCR product was cloned into the *Eco*RI and *Xho*I sites of pET30a (Novagen, Madison, WI, USA) and the recombinant plasmid pET30-σA was confirmed by restriction analysis and nucleotide sequencing. Positive clones were selected for large-scale production and purification. The plasmid pET30-σA was then transformed into BL21 (DE3) (Invitrogen, CA, USA). The expressed His-σA proteins was purified by using Ni-NTA kit (Qiagen, Valencia, CA, USA). To get rid of other proteins, the expressed recombinant proteins were eluted two or three times. First, 20 mM imidazole was used to elute the impurity proteins, then 60 mM or 100 mM was used to wash out the target proteins directly. The amount of σA proteins in the crude extracts was quantified by the DC protein assay (Bio-Rad Laboratories, Tokyo, Japan).

### 2.3. Monoclonal Antibodies Production and Coupling to Horseradish Peroxidase

The purified His-σA proteins were used as antigen to immunize BALB/C mice and MAbs were developed using techniques similar to that described previously [30]. Briefly, spleens were removed from mice immunized with His-σA proteins. Splenocytes were fused with NS1 myeloma cells. Hybridoma cell lines secreting antibodies against His-σA were screened in an indirect ELISA as described for antibody binding assay. Antibodies that bound to His-σA protein but failed to bind His-tag σB protein (His-σB) (as negative control) [5] were considered to be positive. Each hybridoma cell lines subcloned four times and ascitic fluids were prepared with the cloned hybridoma in BALB/C mice. Clonotypes of the obtained MAbs were determined by SBA Mouse Immunoglobulin Clonotyping System-HRP (SouthernBiotech Association, Inc., Miami, FL, USA) according to the manufacturer’s instruction. Immunoglobulins fractions were isolated with saturated ammonium sulfate (pH 7.0) as described previously [24] and then purified with an affinity column of protein G-agarose (Boehringer Mannheim, Inc., Baden-Württemberg, Germany). Purified immunoglobulins were coupled to HRP by the periodate method as described previously [36].

### 2.4. Characterization of MAbs by Western Blot, Immunofluorescence, and Dot Blotting Assays

Purified His-σA were prepared either by dilution with TNE buffer (0.01 M Tris–HCl, 0.1 M NaCl, 0.001 M EDTA, pH 7.4) or by boiling for 5 min in a solution containing 2% SDS and 5% 2-mercaptoethanol. Purified His-σA protein was subjected to 10% SDS-PAGE and transferred to nitrocellulose membranes. The membranes were probed with different MAbs followed by a secondary HRP-conjugated goat anti-mouse antibody (KPL, MD, USA). For immunofluorescence assay, BHK-21 cells infected with DRV (5 M.O.I.) in six-well plates were fixed with cold methanol and then probed with anti-σA MAbs or normal mouse serum (as negative control). Bound antibodies were detected using fluorescent conjugated antibodies against mouse IgG (1:500 dilutions) (KPL, Gaithersburg, MD, USA) under a fluorescence microscope. For dot blotting assay, the same above prepared His-σA was used. Briefly, approximately 1 μg His-σA or His proteins (as negative control) were spotted onto nitrocellulose membrane. The membranes were probed with the MAbs similar as for Western blot.

### 2.5. Antibody Binding Assay and Competitive Binding Assay (CBA)

The amount of binding uncoupled or HRP-coupled antibodies in the ELISA was determined as described previously [30,36,37]. Briefly, for uncoupled MAb determination, ELISA plates were coated with purified His-σA and washed with a TNE buffer containing 2% BSA. The plates were incubated with serial diluted purified MAb. After washing, the plates were detected with HRP-conjugated goat anti-mouse IgG serum (1:500 dilution). The enzymatic activity was determined by absorbance at 405 nm. For HRP-conjugated MAb determination, the same procedures were carried out except that HRP-conjugated MAbs were directly added to the antigen coated plates without using the HRP-conjugated goat anti mouse IgG. The highest binding level and the MAb concentration at which 50% binding were obtained from the resulting dose-response curve. CBA were conducted as described previously [30]. The degree of competitive binding was measured at 405 nm in the presence or absence of unconjugated competing antibodies. Competition was rated as significant strong (++) if it was more than 60%, strong (+) if it was more than 30%, and negative (−) if it was less than 30%.

### 2.6. Epitope Mapping

Purified MAbs 1A7 and 4E2 were selected for epitope mapping under three rounds of biopanning with the Ph.D-12 TM Phage Display Peptide Library Kit (New England BioLabs, Cambridge, MA, USA), as previously described [33]. Briefly, 96-well plates were coated with purified MAbs and incubated with blocking buffer. The phage library was then added to the plate and incubated for 1 h. After five washes with TBS buffer, 1 M Tris-HCl was added to the plate to elute the bound phages. The phages were then amplified and titred on LB/IPTG/Xgal plates for selection. The ratio of output to input was calculated as the titre of the amplified output phages to the titer of the input phages.

### 2.7. Sequence Analysis

To demonstrate the level of conservation of the epitope sequence regions, we constructed sequence alignments of the epitope regions in the corresponding locations in the σA proteins among the representative DRV and ARV using the DNASTAR Lasergene program (DNASTAR Inc., Madison, WI, USA) [38]. Information of representative DRV and ARV from GenBank was listed in Table S1.

### 2.8. Cross-Reactivity of the Epitopes

The epitope reactions to sera against DRV and ARV were determined by the dot blotting assay. Approximately 1 µg of each synthesized epitope peptides (listed in Table 1) or the control peptide GST-YAEYI (epitope of duck Tembusu virus E protein) [34] diluted with TNE buffer were spotted onto the NC. Then, the NC membranes were incubated with sera against DRV or ARV at 37 °C for 1 h. After three washes with PBST, the NC membranes were probed with a corresponding HRP-conjugated IgG or IgY (KPL) at 37 °C for 1 h.

### 2.9. Competitive Inhibition Binding Assay

To test recombinant GST-EAPYPG or GST-WVVAGLI peptide inhibition of corresponding MAbs 4E2 and 1A7 binding to the His-σA protein, the procedures of competitive inhibition binding assay were conducted. Briefly, 100 µL σA antigen (twofold diluted) was coated in ELISA plates and then blocked with 1% BSA. The recombinant GST-EAPYPG or GST-WVVAGLI or the unrelated control peptide GST-YAEYI [33] (diluted in PBST) was mixed with the MAb 4E2 or 1A7. The mixtures of peptide/antibody were added to the σA coated plates. After washing, HRP-conjugated goat anti-mouse IgG was added, and the competitive inhibition binding activity was assessed. The mean optical density at 405 nm (OD405) plus three times the standard deviation was used to determine the cutoff value.

### 2.10. Epitope Peptides for Detection of DRV and ARV Infections

The ELISA procedures for detection the reactivity of the GST-EAPYPG and GST-WVVAGLI peptides to DRV- or ARV-positive sera were similar to those that have been described previously [34]. Briefly, ELISA plates were coated with 5 µg/mL of each synthetic peptide antigen. After washing with PBST, the plates were blocked with PBST containing 5% (*w*/*v*) skimmed milk at 4 °C over-night. The diluted DRV-positive/negative sera in twofold dilutions starting from 1:100 dilution or ARV-positive/negative sera diluted in blocking solution were added and incubated for 1 h at 37 °C. After washing, the corresponding goat anti-duck IgG or goat anti-chicken IgY conjugate (1:500 dilution) were added and incubated at 37 °C for 45 min. After washing, the plates were incubated with p-nitrophenyl phosphate (PNPP) substrate (Shanghai Biomedicine, Shanghai, China) and then the reactions were stopped by 3 M NaOH. OD405 were measured using ELISA reader (Bio-Rad, Beijing, China).

## 3. Results

### 3.1. σA Protein Expression

**Figure 1:**
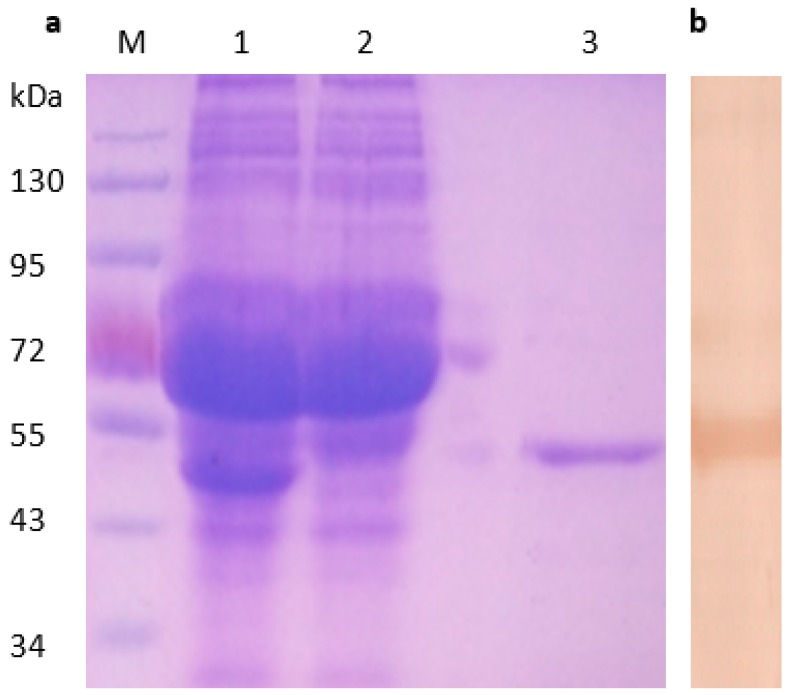
Identification of recombinant His-σA protein from transformed *E. coli* cells. SDS-PAGE analysis of expressed His-σA protein from transformed *E. coli* cells (**a**). Lane M, molecular weight marker; lane 1 and 2, lysate precipitate *E. coli* transformed with plasmid pET30-σA; lane 3, purified His-σA protein; Purified recombinant His-σA protein detected by Western blot with duck anti-DRV serum (**b**).

The σA-encoding gene of DRV was amplified with primers pAF/pAR, and resulted in expected sizes of 1248 bp [7]. The amplified PCR products were cloned into pET30a-σA expression plasmids as described previously [5]. The extracts of *E. coli* transformed with pET30a-σA were analyzed by SDS-PAGE (10% polyacrylamide), and revealed the presence of fusion His-σA protein approximately 55 kDa (Figure 1a), which were consistent with the expected size of His-σA fusion protein. The expressed His-σA fusion proteins were then purified with an Ni-NTA kit (Qiagen, Valencia, CA, USA). The total amount of proteins in the crude extracts was quantified by the DC protein assay (Bio-Rad). The purified His-σA protein was then detected with duck anti-DRV polyclonal serum (Figure 1b). Western blot analysis showed that purified His-σA proteins reacted specifically with duck anti-DRV polyclonal antibody with an approximate molecular mass of 55 kDa, indicating that recombinant His-σA protein was successfully expressed. 

### 3.2. Characterization of MAbs

**Figure 2:**
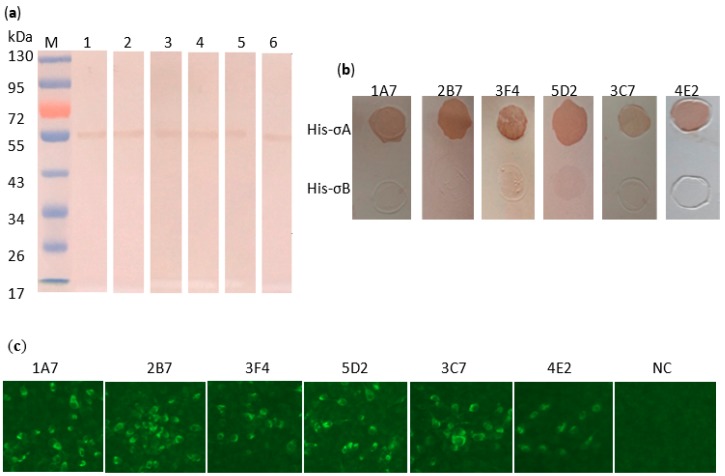
Characterization of anti-σA MAbs of DRV. Detection of expressed recombinant His-σA protein by Western blot with MAbs (**a**). Lane 1, MAb 1A7; lane 2, MAb 2B7; lane 3, MAb 3F4; lane 4, MAb 5D2; lane 5, MAb 3C7; lane 6, MAb 4E2. Detection of σA protein with mAbs in BHK-21 cells infected with DRV by indirect immunofluorescence assay (**b**). No specific fluorescence was found for uninfected cells (400×). Detection of expressed recombinant His-σA or His proteins with anti-σA mAbs by Dot blotting assays (**c**).

Six hybridomas cell lines secreting anti-σA antibody were obtained after four rounds of subcloning. The isotypes of MAbs were IgG1 (1A7, 3F4, 5D2, 4E2) and IgG2b (3C7 and 2B7), respectively. The function of the conformation of His-σA in MAbs binding activity was characterized by Western blot and dot blotting analyses. All MAbs showed binding activities to His-σA in their native conformation, i.e., in TNE buffer (Figure 2a,b). Six MAbs were divided into three epitope groups (named I, II, and III): epitope I include 1A7, 2B7, 3F4, epitope II only 5D2, and III include 3C7 and 4E2 (Table 1). When the denatured His-σA protein by SDS and 2-mercaptoethanol was probed with MAbs, the binding of MAb 5D2 recognizing epitope II was completely abolished (data not shown). The results indicate that recognition of MAb 5D2 to epitope II required the native conformation of σA, suggesting that its binding activity was conformation-dependent. While epitopes I and III on σA proteins were resistant to the SDS and 2-mercaptoethanol treatment, confirming that binding activities of MAbs to epitopes I and III were conformation-independent. All MAbs did not react with His proteins no matter whether they were treated by SDS and 2-mercaptoethanol or not, confirming that MAbs were specific to σA protein. An immunofluorescence assay (IFA) was also used to assess whether the MAbs recognize the native form of σA protein in virus infected cells. IFA showed that six anti-σA MAbs reacted with DRV infected BHK-21 cells, while uninfected cells showed no fluorescence signal (Figure 2c), which indicated that MAbs were specifically anti-σA.

### 3.3. Competitive Binding Assay

The proper concentrations for the competitive binding assay were determined using dose-response curves plotted for unconjugated and HRP-conjugated MAbs (data not shown). Each of the six MAbs was used both as a competitor and as an HRP-conjugated probe. The percentage of competition was normally 100% in the presence of a saturating unlabeled homologous antibody. Three distinct epitopes on σA were found and designated I, II, and III (Table 1). 1A7, 2B7, and 3F4 belong to epitope I, 5D2 belong to epitope II, and 3C7 and 4E2 belong to epitope III.

### 3.4. Epitope Mapping

**Figure 3:**
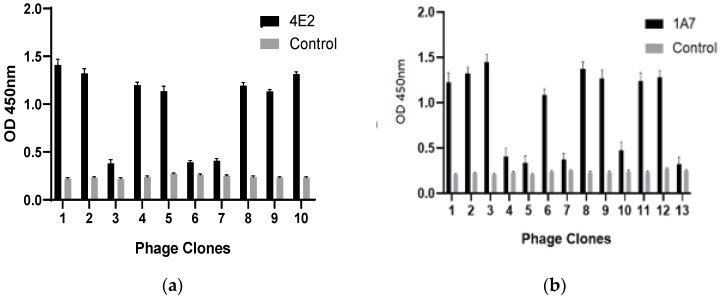
Reactivity of phage clones to MAbs 4E2 and 1A7 binding in enzyme-linked immunosorbent assay (ELISA). The selected phage clones were detected by MAb 4E2 (**a**) and 1A7 (**b**) or the anti-porcine interferon (IFN)-MAb (negative control) after three rounds of biopanning. OD, optical density.

A 12-mer peptide phage library was used to screen epitopes by MAbs 1A7 and 4E2. After three rounds of biopanning, the reactivities of phage clones to MAbs 1A7 and 4E2 were evaluated. Seven of ten clones reacted with 4E2, while eight of thirteen clones reacted with MAb 1A7 (OD450 nm ≥1.10) and the rest of the clones were less reactive (OD450 nm <0.41) (Figure 3a,b). None of the selected clones reacted with the anti-porcine IFN-c mAb (OD450 nm <0.27). Sequencing of the phage clones with high OD values revealed the consensus sequences EAPYPG and WVV/MAGLI/V (Table 2), which were identical to the ^56^EAPYPG^61^ (aa 217 to 223) and ^341^WVVAGLI^347^ (aa 341–347) sequences of the DRV σA protein.

### 3.5. Sequence Analysis of the Identified Epitopes Among DRV and ARV

**Figure 4:**
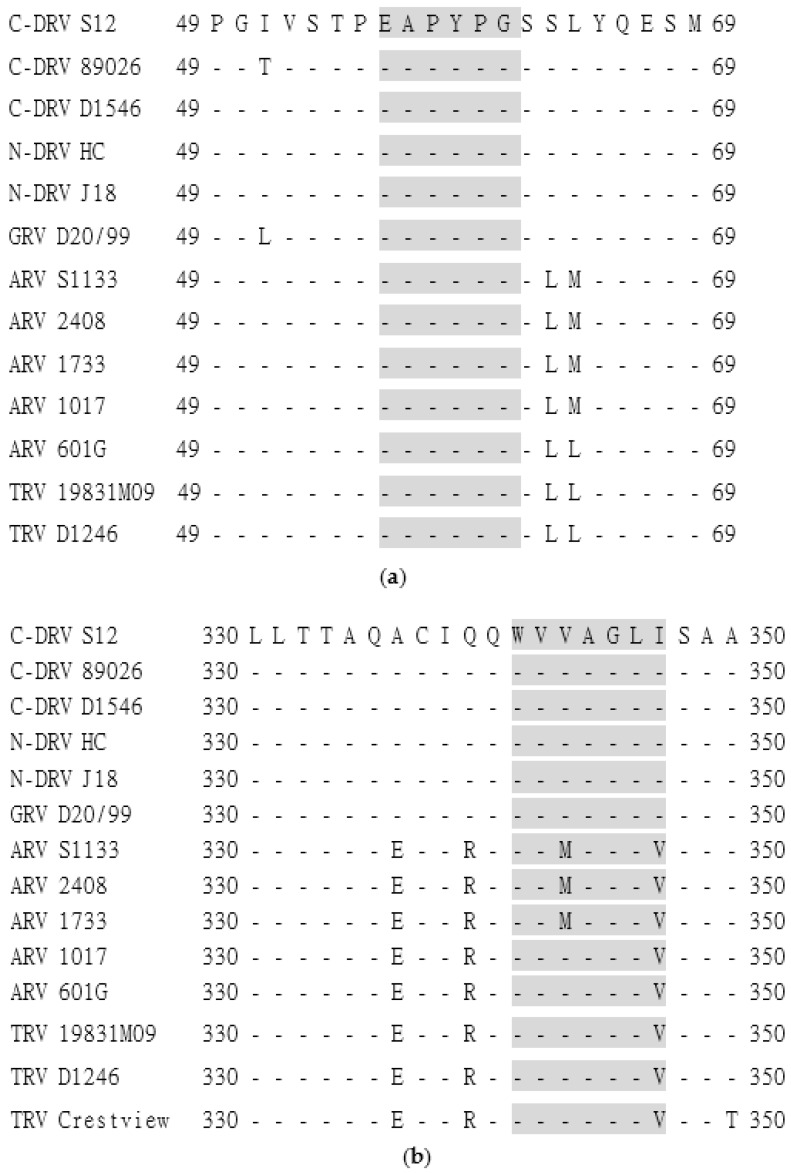
Sequence alignments of the epitope EAPYPG (**a**) and WVVAGLI (**b**) regions in the σA protein of DRV, GRV, ARV, and TRV. The amino acid positions for each sequence are numbered on both sides. The C-DRV S12 strain sequence is shown on the top and the differences were indicated. The dashes indicate identical amino acids.

The conservation of epitopes in the σA were determined by alignment of the epitope sequences of DRV, GRV, ARV, and TRV (**Supplementary** listed in Table S1). Residues in epitope ^56^EAPYPG^61^ were completely homologous in DRV, GRV, ARV, and TRV (Figure 4a), while residues in the ^341^WVVAGLI^347^ were homologous in DRV and GRV, but divergent at residues ^343^V/M or/and ^347^I/V between DRV/GRV and ARV/TRV (Figure 4b).

### 3.6. Fine Mapping of the Epitopes by Western Blot

**Figure 5:**
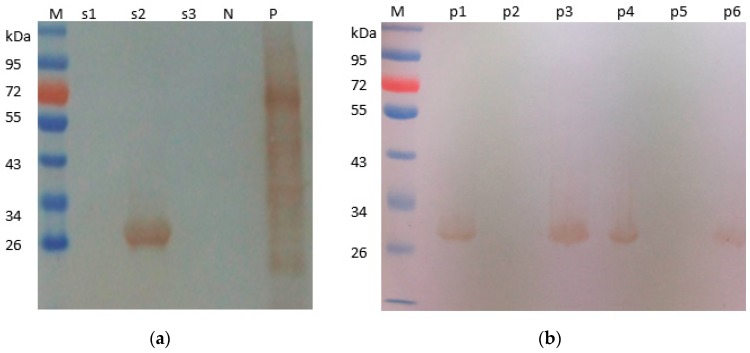
Fine mapping epitopes EAPYPG and WVV/MAGLI/V by Western blot. GST-fusion polypeptides containing truncated motif derived from sequence EAPYPG were detected with MAb 4E2 (**a**). GST-σA and peptide GST-YAEYI were used as positive and negative control, respectively. GST-fusion polypeptides containing truncated or muted motif derived from sequence WVV/MAGLI/V were detected with MAb 1A7 (**b**). The GST-fusion polypeptides are listed in Table 3.

To determine the minimal epitopes, mutated peptides based on core motif EAPYPG or WVVAGLI were expressed and detected by Western blot with corresponding 4E2 or 1A7. Figure 5a showed that only GST-EAPYPG and GST-σA could reacted with MAb 4E2, but GST-APYPG or GST-EAPYP or negative control peptide did not react, suggesting that EAPYPG is the minimal epitope and residues ^56^E and ^61^G are crucial in binding. To determine whether ^343^M or/and ^347^V in WVV/MAGLI/V were crucial residues, expressed mutated GST-WVVAGLI fragments were detected by MAb 1A7 by Western blot. Figure 5b showed that residue substitution of ^343^V with ^343^M or/and ^343^I with ^347^V in ^343^WVV/MAGLI/V^347^ did not abolish 1A7 binding activity, suggesting that ^343^M or/and ^347^V in ^343^WVV/MAGLI/V^347^ were mutual replaceable residues at this position. Deletions of residue ^341^W or ^347^I at N- or C-terminus of ^341^WVV/MAGLI/V^347^ lost 1A7 binding activity, suggesting that WVV/MAGLI/V was the minimal epitope recognized by 1A7.

### 3.7. Epitopes Cross-Reactivity to DRV and ARV-Positive Sera

**Figure 6:**
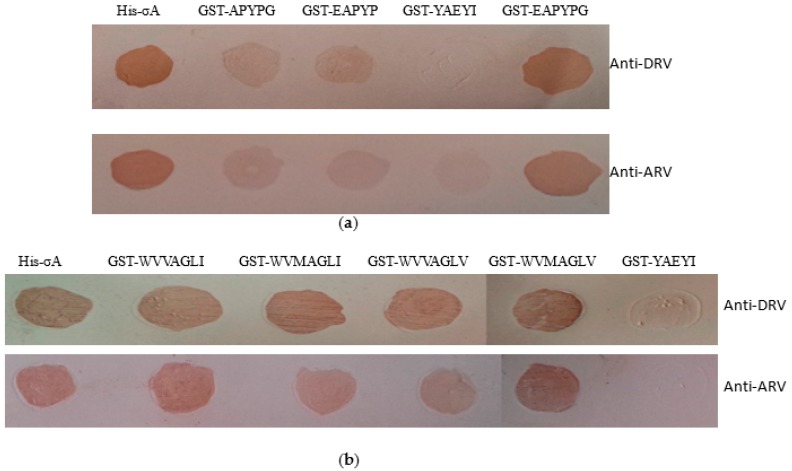
The cross-reactivity of the epitope peptides GST-EAPYPG (**a**) and GST-WVVAGLI (**b**) to DRV- and ARV-positive serum in the dot blotting assay. GST-YAEYI and the GST-σA protein were used as the negative and positive controls, respectively.

To evaluate the epitopes’ EAPYPG and WVV/MAGLI/V reactivity to DRV and ARV, a dot blotting assay was used to test each polypeptide fragments (listed in Table 3) binding ability to DRV and ARV-positive sera. Figure 6 showed that GST-EAPYPG or GST-WVV/MAGLI/V or GST-σA could react with both DRV and ARV-positive serum, but control peptide GST-YAEYI did not show any reaction to the DRV and ARV-positive serum (Figure 6). We did not evaluate epitope reactivities to GRV and TRV, because of unavailable GRV- and TRV-positive sera.

### 3.8. Competitive Inhibition of Recombinant Peptides Binding to MAbs 1A7 and 4E2

**Figure 7:**
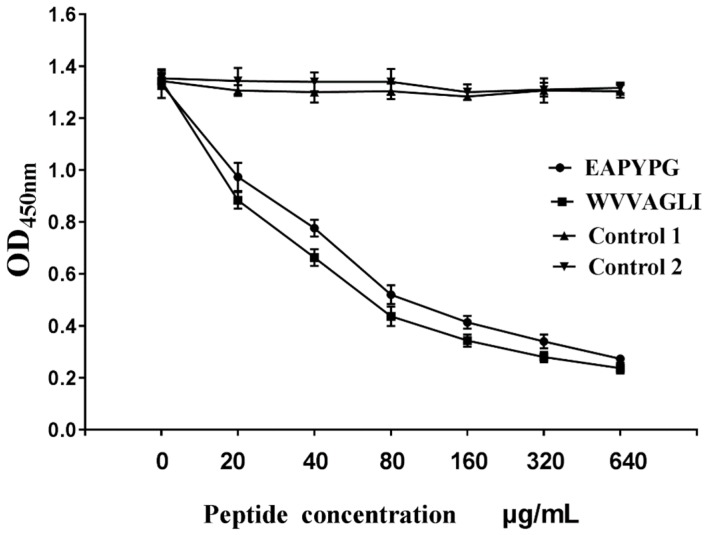
Competitive inhibition of recombinant peptides GST-EAPYPG and GST-WVVAGLI binding to corresponding MAb 4E2 and 1A7. A competitive inhibition ELISA was conducted using the antigen peptide GST-EAPYPG or GST-WVVAGLI as the competitor for σA protein. Data represents three independent experiments with triplicates included in each experiment (*p* < 0.05).

To confirm that peptides GST-EAPYPG and GST-WVVAGLI were epitopes of 4E2 and 1A7, competitive binding inhibition assays were performed. These assays showed that the reactivity of 4E2 and 1A7 with σA protein was inhibited by the corresponding antigen peptides GST-EAPYPG and GST-WVVAGLI in a dose-dependent manner (*p* < 0.05, Figure 7). The control peptide GST-YAEYI showed no inhibition.

### 3.9. An Epitope-Based Peptide ELISA for Diagnosis of DRV and ARV

**Figure 8:**
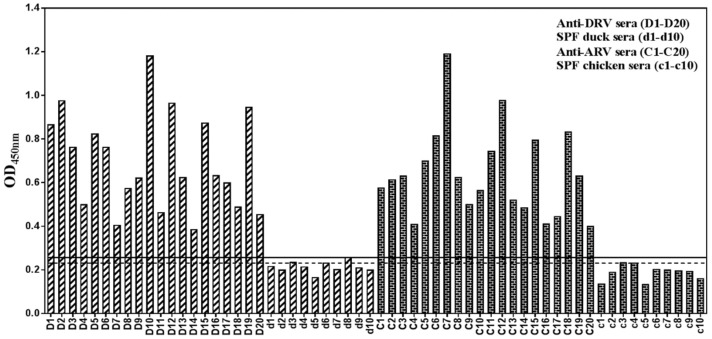
ELISA reactivity of the synthetic epitope peptides against serum samples. D1–D20: 20 serum samples from DRV infected ducks; d1–d10: 10 serum samples from PBS infected SPF ducks (as negative control); C1–C20: 20 serum samples from ARV infected chickens. c1–c10: 10 serum samples from PBS infected SPF chickens (as negative control). The cutoff values (solid line for DRV and dashed line for ARV detection) were calculated as 0.256 and 0.243.

Mean OD405 nm plus three times the standard deviation were used to determine the cutoff values. The cutoff values were 0.256 (0.2151 + 3 × 0.0138) and 0.243 (0.2047c + 3 × 0.0128) for detection of DRV and ARV infection, respectively. With combined peptides GST-EAPYPG/GST-WVVAGLI as antigen, the ELISA was able to detect 19 DRV and 20 ARV infections in 20 serum samples collected from 20 duck or chicken experimentally infected with DRV or ARV. In contrast, ten control SPF duck or ten SPF chicken sera were seronegative using the same procedure (Figure 8). The other sera against H5N1 influenza virus, New Castle disease virus, Tembusu virus, and parvovirus were also sera-negative using the same epitope-based peptide serologic test. (data not shown). The specificity of this method was 100% for the sera of SPF-duck and SPF-chicken without reovirus infection. The sensitivities of this ELISA procedure were 96% and 100% for DRV and ARV infections, respectively.

## 4. Discussion

Six MAbs were developed by immunizing mice with the expressed His-σA protein of DRV after several rounds of subcloning. Six MAbs were divided into three different epitope binding groups (I, II, and III) after a competitive binding assay. Western blot and dot blotting analyses showed that these MAbs could recognize the expressed His-σA protein but not His proteins. Moreover, an immunofluorescence assay demonstrated that these MAbs could bind the authentic native viral σA protein in DRV-infected cells, confirming that the epitopes recognized by these MAbs were present on the viral σA protein. Five of six MAbs recognized denatured His-σA protein, but MAb 5D2 lost its binding activity when His-σA protein was treated with SDS and 2-mercaptoethanol, suggesting that all MAbs except 2D5 are conformation-independent.

Using a phage display system and mutagenesis, we precisely mapped the minimal B-cell epitopes to two locations, amino acids 56–61 and 341–347 of the σA protein. Dot blotting and Western blot analyses with MAb 1A7 and 4E2 and N- or C-terminal deletion mutants of the epitopes demonstrated that the motifs EAPYPG and WVVAGLI were the minimal units required for maximal binding activity. Moreover, the competitive inhibition assay of 1A7 binding to synthetic WVVAGLI or 4E2 binding to EAPYPG and to His-σA protein fragments verified that EAPYPG and WVVAGLI were epitopes of the σA protein.

The results that EAPYPG sequence is completely similar or residues ^343^V/M and ^347^I/V are replaceable in WVV/MAGLI/V for both DRV and ARV, and their cross-reactivities to both DRV and ARV-positive sera, confirmed that EAPYPG and WVVAGLI might be the minimal cross-reactive epitopes for both of DRV and ARV. Moreover, with a different epitope mapping technique, a similar epitope 340 QWVMAGLVSAA 350 was identified with MAb against σA of ARV, confirming that the epitope at position 341–347 should be a cross-reactive epitope [39]. Further experiments are necessary to conduct for the functional role of this epitope.

By taking the advantages of the specificity of epitope peptides, an ELISA based on epitope peptides as antigens was developed to detect σA-specific antibodies in DRV and ARV-infected birds. The epitope ELISA clearly differentiates serum samples between the DRV-/ARV- and uninfected birds as indicated by absorbance, suggested that recombinant GST-EAPYPG and GST-WVV/MAGLI/V peptides were useful antigens for detection of DRV and ARV infections. The epitope ELISA is relatively simple and specific and does not require the paired serum samples needed for conventional tests. Further study on the application of these two epitope-based ELISA with a large number of clinical samples should be conducted.

## 5. Conclusions

In summary, we developed and characterized six MAbs and identified for the first time two minimal cross-reactive epitopes of the σA protein of bird reoviruses. This information will provide new insights into the structure and organization of epitopes on the σA protein of bird reoviruses and valuable cross-reactive epitope information for the development of epitopes-ELISA diagnostic assays for the detection of DRV and ARV infections.

## Figures and Tables

**Table 1:** Results of competitive binding assay between MAbs.

Competitor	Clone Type		HRP-Labled Mab					
			1A7	2B7	3F4	2D5	3C7	4E2
Epitope I	1A7	IgG1	**++**	**++**	**++**	**−−**	**−−**	**−−**
	2B7	IgG2b	**++**	**++**	**++**	**−−**	**−−**	**−−**
	3F4	IgG1	**++**	**++**	**++**	**−−**	**−−**	**−−**
Epitope II	2D5	IgG1	**−−**	**−−**	**−−**	**++**	**−−**	**−−**
Epitope III	3C7	IgG2b	**−−**	**−−**	**−−**	**−−**	**++**	**++**
	4E2	IgG1	**−−**	**−−**	**−−**	**−−**	**++**	**++**

**Table 2:** Peptide sequences of the selected phage clones.

Phage Clones	Sequences															Phage Clones	Sequences													
1	T	M	A	N	**E**	**A**	**P**	**Y**	**P**	**G**	T	Q				1	A	F	**W**	S	**V**	P	G	T	**V**	S	M	T		
2			H	W	**D**	**P**	**P**	**Y**	**P**	**G**	S	G	T	Q		2		N	**W**	V	**V**	G	**G**	S	**V**	A	A	V	T	
4				T	**E**	**A**	**P**	**Y**	**P**	**G**	S	S	V	T	N	3	Y	N	F	**V**	**M**	**A**	**G**	**L**	**V**	A	T	T		
5	S	A	L	I	**D**	**A**	**A**	**Y**	**P**	**G**	T	Q				6		I	**W**	**V**	**M**	**A**	**G**	A	**I**	S	L	S	M	
8				S	**E**	**A**	**P**	**Y**	**P**	**G**	A	G	V	Y	N	8			Y	**V**	**M**	**A**	**G**	**L**	**I**	L	S	I	P	N
9			T	F	**E**	**A**	**P**	**Y**	**P**	**G**	T	M	L	V		9		C	F	T	**M**	**A**	S	**L**	**I**	T	I	T	A	
10			S	T	**E**	**A**	**P**	**Y**	**P**	**G**	I	A	V	Q		11	G	A	**W**	**V**	**V**	**A**	**G**	**L**	**I**	M	T	V		
Consensus					**E**	**A**	**P**	**Y**	**P**	**G**						12		Q	**W**	**V**	**V**	**A**	**G**	**L**	**V**	S	V	L	G	
DRV	V	S	T	P	E	A	P	Y	P	G	S	S	L	Y	Q	Consensus			**W**	**V**	**V/M**	**A**	**G**	**L**	**I/V**					
																DRV	Q	Q	W	V	V	A	G	L	I	S	A	A	K	G

**Table 3:** Primers for the mutated epitope fragments.

Peptide	Primers	Sequences	Muted Epitope Peptide
S1	pSF1	5-aattc gag gcg cct tac ccg ggc c-3	GST-EAPYPG
	pSR1	5-tcgag gcc cgg gta agg cgc ctc g-3	
S2	pSF2	5-aattc gcg cct tac ccg ggc c-3	GST-APYPG
	pSR2	5-tcgag gcc cgg gta agg cgc g-3	
S3	pSF3	5-aattc gag gcg cct tac ccg c-3	GST-EAPYP
	pSR3	5-tcgag cgg gta agg cgc ctc g-3	
S4	pSF4	5-aattc gcg gcg cct tac ccg ggc c-3	GST-AAPYPG
	pSR4	5-tcgag gcc cgg gta agg cgc cgc g-3	
S5	pSF5	5-aattc gag gcg cct tac ccg gcg c-3	GST-EAPYPA
	pSR5	5-tcgag cgc cgg gta agg cgc ctc g-3	
P1	pF1	5-aattc tgg gtc gtg gct ggt ctg att c-3	GST-WVVAGLI
	pR1	5-tcgag aat cag acc agc cac gac cca g-3	
P2	pF4	5-aattc gtc gtg gct ggt ctg att c-3	GST-VVAGLI
	pR4	5-tcgag aat cag acc agc cac gac g-3	
P3	pF3	5-aattc tgg gtc atg gct ggt ctg att c-3	GST-WVMAGLI
	pR3	5-tcgag aat cag acc agc cat gac cca g-3	
P4	pF3	5-aattc tgg gtc gtg gct ggt ctg gtg c-3	GST-WVVAGLV
	pR3	5-tcgag cac cag acc agc cac gac cca g-3	
P5	pF5	5-aattc tgg gtc gtg gct ggt ctg c-3	GST-WVVAGL
	pR5	5-tcgag cag acc agc cac gac cca g-3	
P6	pF6	5-aattc tgg gtc atg gct ggt ctg gtg c-3	GST-WVMAGLV
	pR6	5-tcgag cac cag acc agc cat gac cca g-3	

## Supplementary Materials

The following are available online at https://www.mdpi.com/2076-0817/8/3/140/s1, Table S1. Duck and avian reovirus strains used for the sigma A sequence analysis in this study.
