# Supplementary material for: Characterization of Monoclonal Antibodies against σA Protein and Cross-Reactive Epitope Identification and Application for Detection of Duck and Chicken Reovirus Infections
Source: Pathogens. 2019 Sep 7;8(3):140. doi: 10.3390/pathogens8030140 (PMC6789564; doi:10.3390/pathogens8030140)
Supplement: Supplementary file 1 [file pathogens-08-00140-s001.pdf]

Table S1. Duck and avian reovirus strains used for the sigma A sequence analysis in this study.

Species	Strain	GenBank No.	Location	Year of isolation
C-DRV	S12	EF076764	China	2006
C-DRV	89026	AJ278102	France	2002
C-DRV	D-1546	KJ871023	France	2014
N-DRV	J18	JX478267	China	2008
N-DRV	HC	FJ858376	China	2009
GRV	D20/99	KF809669	Hungary	1999
ARV	S1133	AF104311	Taiwan	1998
ARV	1733	AF293773	Taiwan	2000
ARV	2408	AF247724	Taiwan	2000
ARV	1017	AF294762	Taiwan	2000
ARV	601G	AF311322	Taiwan	2000
TRV	Crestview	KF872244	USA	2011
TRV	19831M09	KR997906	Hungary	2015
TRV	D1246	KR997916	Hungary	2015
